# An Unbelievable Fact on “Dialysis Centers Without Hepatitis C Infection”

**DOI:** 10.5812/numonthly.17998

**Published:** 2014-03-01

**Authors:** Seyed Moayed Alavian

**Affiliations:** 1 Baqiyatallah Research Center for Gastroenterology and Liver Diseases, Baqiyatallah University of Medical Sciences, Tehran, IR Iran; 2 Middle East Liver Disease Center, Tehran, IR Iran

**Keywords:** Hepatitis C, Renal Dialysis, Epidemiology

Hepatitis C virus (HCV) infection is the second viral cause for chronic liver disease (CLD) in the world and nearly 170 million people are infected worldwide (1, 2). The history of transfusion before 1992 (time of blood and blood products screening), illegal drug using, exposures among health care workers, unprotected multi-partner sexual contact, and chronic hemodialysis are known risk factors for HCV infection (3-5). HCV infection is a common infection in hemodialysis (HD) patients as well as dialysis centers (6). It is currently the major cause of CLD and mortality after kidney transplantation (7). Distribution of HCV infection among HD patients is not globally homogenous. We performed a multicenter study in Tehran in 2002 and the prevalence of HCV infection among HD patients was around 13% (8). We systematically reviewed all published and unpublished documents related to HCV infection prevalence in Iranian HD patients from April 2001 to March 2008 (9). Eighteen studies from 12 provinces (consisting of 49.02% of the total Iranian population) reported prevalence of HCV infection in Iranian HD patients. HCV infection prevalence in Iranian HD patients was 7.61% and showing that the burden has changed during the recent years (9).

In 2006, we started a project for control of hepatitis C infection in dialysis patients (10). Iran Hepatitis Network (IHN), Ministry of Health and Medical Education (MOHME), many experts in hemodialysis and gastroenterology and infectious departments in many universities were involved in it in Iran. We presented main strategies for control of HCV infection in dialysis centers that consisted of: periodic screening of HCV in dialysis centers and reporting to MOHME for evaluation, distributing information to both patients and health staffs, training courses and congresses in various cities with more focus on the cities with higher HCV infection prevalence, treatment of HCV-positive patients with approved protocols, treatment according to a widely approved protocol, and putting HCV-positive HD patients in the top of waiting list for kidney transplantation (10). Changes in the epidemiology of HCV infection and decrease in the burden of liver diseases in HD patients has been reported according to the report of MOHME in Iran (11). The prevalence of HCV has decreased from 14.4% in 1999 to 4.5% in 2006 (11). Recently published articles of different centers from various parts of Iran showed the continuation of this burden decrease. Samimi-Rad et al. reported the prevalence of HCV antibody among HD patients around 5.0% in Yazd province and 5.4% in Markazi province (12, 13). Fortunately, the authors performed the molecular tests for all anti-HCV antibody-positive cases in their study. Zahedi et al. reported the prevalence of HCV antibody among HD patients around 7% in Kerman province (14) and Assarehzadegan et al. reported it 7.9% in Khuzestan province (15). In Zahedi et al. study, less than 50% of positive cases were HCV RNA-positive, meaning that less than 3.5% of their samples were HCV infected. I would like to mention that we should evaluate the epidemiology of HCV infection in addition to serological tests, using molecular tests such as RT-PCR, and ask the history of antiviral therapy in hemodialysis patients with anti-HCV Ab-positive results and consider the RT-PCR negative results as cured cases, not positive ones. This mistake might result in over-estimation of the prevalence of HCV infection in this high group. antiviral therapy; introduction of erythropoietin which has resulted in the decrease of transfusion needs; early transplantation; treatment of positive cases; training health staffs; and higher mortality of HD patient with HCV infection on hemodialysis. Finally, I would like to emphasize that we can imagine the “dialysis center without HCV” if we follow the standard infection precautions for control of HCV infection.
